# Desiccation tolerant yet short-lived seeds: A conundrum for post-harvest handling of a high restoration value bunchgrass?

**DOI:** 10.1371/journal.pone.0326596

**Published:** 2025-06-20

**Authors:** Sarah Tevlin, Héctor E. Pérez, Raelene M. Crandall, Jennifer M. Fill

**Affiliations:** 1 Department of Environmental Horticulture, University of Florida, Gainesville, Florida, United States of America; 2 School of Forest, Fisheries, and Geomatics Sciences, University of Florida, Gainesville, Florida, United States of America; University of Innsbruck, AUSTRIA

## Abstract

The successful preservation of seeds for future use depends on factors that maintain or limit seed viability. Yet, seed biology knowledge that would facilitate seed storage of most wild species used in ecosystem restoration is absent. This study characterized changes in seed water content, germination, desiccation tolerance, and relative storage longevity of *Aristida beyrichiana* (wiregrass), a focal restoration species, with respect to habitat of collection. We collected mature seeds from mesic and xeric habitats over two years then exposed these to desiccation stress sufficient for germplasm storage and aging stress (60% relative humidity, 45°C). We followed each method with germination assays at simulated seasonal temperatures. We analyzed germination responses along with production of normal and abnormal seedlings. We then modeled potential seed longevity and compared this against longevity of reference species. We found that wiregrass seeds display sufficient desiccation tolerance for ex situ storage and germinate preferentially under spring or fall and summer temperature conditions. The negligible to small effects of ecotype on these responses do not support the hypothesis that habitat of occurrence represents an adequate predictor of desiccation tolerance or germination response. However, seeds from both xeric and mesic habitats are estimated to be short-lived in storage. The contradiction between high desiccation tolerance yet short-lived nature of seeds implies that proper post-harvest seed handling, particularly regarding seed moisture management, is critical for maintaining seed viability. Further implications of this interesting seed physiology are discussed in the context of restoration.

## Introduction

The successful preservation of seeds for future use in ecological restoration programs depends on knowledge of factors that maintain or limit seed viability. Such seed longevity information is beneficial because seeds intended for plant restoration programs are rarely sown immediately after harvest. Stored seeds can be a critical source of propagules during periods when, or for species which, seeds are only sporadically available. Moreover, seed-sourcing guidelines often require considering ecotypic adaptation between donor and restoration sites [1–4]. Having adequate genetic representation in germplasm storage thereby facilitates site matching. Accordingly, ex situ seed storage is an indispensable component of global land rehabilitation efforts [5].

Seed longevity is a dichotomous concept. Along one branch, longevity describes seed lifespan during ex situ storage. This type of storage is often associated with facilities that can precisely control temperature and humidity. Longevity in this context is predictable based on seed moisture content and temperature during storage [6,7]. The ability to control these factors has immense implications for plant restoration programs [5,8,9]. Along the other branch, longevity represents the capacity of seeds to maintain viability in the soil seed bank. Soil seed banks provide a buffer against environmental fluctuations to ensure species persistence and, therefore, have considerable ecological importance [10,11].

A primary goal of ex situ storage is to limit the extent and rate of deleterious aging reactions. Practitioners accomplish this by first desiccating seeds to about 3–12% moisture content [12–14]. Drying to this level is achieved by manipulating the relative humidity (RH) of air around seeds to 10–25% at 5–20°C [15]. This corresponds to seed water potentials of about −177 to −311 MPa [16]. Depending on facility capabilities and goals, dried seeds are typically sealed in air-tight packaging then stored at low temperatures [15]. This contrasts markedly with field conditions, where soil physical and chemical properties, amount of rainfall or irrigation, temperature changes, water vapor at the air-soil interface, and surrounding vegetation influence soil water potential [17]. Soil water potential in the top 15 cm of the profile fluctuates considerably from about −0.01 MPa at field capacity to less than −1.5 MPa [17]. At one extreme, the near surface water potential of some sparsely vegetated desert soils decreased to about −15.0 MPa, and measurements below −4.0 MPa were common [18–20].

The difference in hydration levels between seeds in ex situ storage and those in the soil is crucial. For example, the cellular cytoplasm of seeds dried to ex situ standards is in an extremely viscous glassy state which restricts molecular mobility. Likewise, respiratory metabolism is inhibited. These mechanisms, among others, render seeds in a nearly homeostatic condition that reduces damage to cellular constituents while limiting oxidative processes that degrade metabolic systems [16,21–23]. Alternatively, the water potential of seeds in the soil bank must generally be greater than −2.0 MPa to complete the germination process [24]. Seeds between about −2.0 to −12.0 MPa remain in a metabolically active yet non-germinated state [23]. These hydration levels allow for physiological processes that repair accumulated aging damage [23] resulting from repeated wet-dry cycles and other environmental stressors. Consequently, a hydration-mediated active repair system is key for seed viability maintenance in the soil [10,11]. Ultimately, all seeds, whether in the soil or ex situ facility, will succumb to aging if germination does not occur [11,16].

Desiccation tolerance is, therefore, an important aspect of seed storage biology that influences resistance to aging under ex situ conditions. Desiccation tolerance refers to mechanisms that seeds possess to avoid cellular damage upon water loss [25]. Although this trait has implications for longevity in the soil seed bank, it is more often associated with seed storage physiology [6,7,23]. For example, seeds that possess relatively high desiccation tolerance (e.g., 5–10% moisture content) and for which shelf life can be extended in a dry, cold (e.g., −18°C) state are “orthodox”. Orthodox storage physiology characterizes the seeds of most plant species [26]. Species producing seeds that cannot survive an appreciable amount of desiccation (e.g., about < 20–30% moisture content) and for which dry storage is not possible are “recalcitrant”. Both labels are associated with estimates of ex situ longevity [7,23]. However, seeds of some species do not fall into either category [23,27]. These so-called “intermediate” seeds most often exhibit one of three storage behaviors: (1) desiccation tolerance to lower moisture contents than recalcitrant seeds, but not as low as orthodox; (2) abnormal ex situ storage longevity responses to low (10 to −30°C) temperatures; or (3) short lifespans irrespective of moisture content or storage temperature [28]. Notably, the third category suggests that ex situ seed longevity for some species may not necessarily be coupled with seed desiccation tolerance.

Seed longevity is also influenced by environmental signals other than moisture that govern seed physiological status. Environmental fluctuations in parameters such as temperature stimulate sufficiently hydrated seeds to germinate [29]. Additionally, some evidence suggests associations between phylogeny or geography and longevity [21]. For instance, species from hot, dry environments tend to produce longer-lived seeds while species from warm, wet sites tend to produce more desiccation-sensitive seeds [26,30,31].

However, precisely estimating longevity presents challenges. Direct measurements of ex situ seed longevity require extended periods as it can take years to decades for seeds to reach a viability decline threshold [16,32,33]. Additionally, generating the data necessary for constructing species-specific seed viability constants [6,34] requires large numbers of seeds and investments of time, specialized equipment and expertise, and sufficient labor, which may not be available to ecological restoration organizations. An alternative protocol to estimate ex situ longevity creates aging conditions by exposing seeds to 60% RH and 45°C [32,35,36]. A justification for this system is that the moisture content of seeds adjusted to these conditions will be within the well-defined range established by viability equations used to predict storage longevity [35; but see [21,23] for counter arguments]. Therefore, species can be ranked by the time necessary for 50% of the seeds to lose viability (*p*_50_). Storage longevity can then be estimated by comparing calculated *p*_50_ values of focal species to species whose seed longevity is known [32,37].

We studied the potential ex situ seed longevity of wiregrass (*Aristida beyrichiana*; syn. *A. stricta*, *A*. *stricta* var. *beyrichiana*) a perennial C_4_ bunchgrass native to the North American Coastal Plain (NACP). This species is dominant in pine savanna understories from xeric sandhills to mesic flatwoods [38]. It is considered a foundation species within the threatened longleaf pine ecosystem due to its ability to carry fire [39] and its associations with biodiverse flora [40]. As a result, wiregrass revegetation is a vital component of restoration projects within the NACP [41,42].

Practitioners engaging in seed-based restoration within the NACP face numerous challenges. First, wiregrass seed supply is extremely constrained [41,42]. Sexual reproduction of wiregrass is almost exclusively stimulated by burning during the early to mid-growing season [38,43]. This trait strictly limits seed collection sites to those with recent fire. Next, practitioners are advised to account for ecotypic adaptation by sourcing seeds from donor sites sharing environmental characteristics with restoration sites [44–46]. Wiregrass seed lot quality is also highly variable, with plants producing up to about 50–80% empty seeds and 2–25% of seeds displaying fungal contamination at harvest [47–50]. Furthermore, post-harvest sorting and conditioning practices that improve wiregrass seed lot quality are not reported.

The ability to store wiregrass seeds would greatly expand restoration capacity. Yet, current evidence for *Aristida* indicates that some species are “certainly orthodox” while others are “likely” or “probably orthodox” [51]. Furthermore, we found no studies reporting the extent to which habitat of origin affects wiregrass seed desiccation tolerance or potential storage longevity. Therefore, we examined the: 1) tolerance of wiregrass seeds to appropriate drying conditions for storage, 2) relative ex situ longevity of seeds determined via controlled aging assays, and 3) response of these functional traits in relation to ecotypic variation of source populations [40,45,46]. Finally, we compare our estimates of ex situ longevity with reports of wiregrass soil seed bank longevity.

## Materials and methods

### Seed material and post-harvest processing

We collected wiregrass caryopses, referred to hereafter as seeds, during the natural shedding phase from populations growing in xeric and mesic habitats throughout central and north Florida that received growing season burns the same year. The collection sites used in this study represent the only populations in our region during the time frame of the study that received prescribed fires, were accessible, and from which we were permitted by the appropriate agencies to collect seeds from. Seed collection occurring in mesic sites within the Hal Scott Preserve (S1 Table) was approved by the St. Johns River Water Management District under a special use authorization executed on 01-Dec-2021. Remaining collections occurred on university or private land where permitting was not required by law.

Collections occurred in early December 2021 and 2022. We subsequently labeled seed sources according to ecotype (X = xeric, M = mesic) and collection year as follows: X-21, M01-21, M02-21, X-22, and M-22. M01-21 and M02-21 represent different burn units separated by about 2 km (S1 Table). We hand-stripped seeds from panicles, then pre-screened seeds from all populations for damage, infection, and fill using the enhanced forceps press test [49]. This two-month-long conditioning process resulted in seed lots consisting only of filled seeds with no visible signs of infection or damage. We stored seeds in paper bags. Thus, seeds equilibrated to laboratory temperature (ca. 23–25°C) and relative humidity (30–50%) conditions. A portion of 2021 seeds remained under laboratory conditions for six months without additional post-harvest drying to low water contents to simulate seed sourcing practices observed during ecological restoration activities (S1 Fig).

### Seed water content

We assessed seed water content from the X-21, M01-21, and M02-21 populations after 6 months of laboratory storage by randomly selecting 25 seeds from each lot. We then divided each sample into five replicates of five seeds each. We measured the fresh mass of all replicates gravimetrically, then dried seeds in a forced air oven (0V-510A-2, Blue M Electric Company, Blue Island, IL, U.S.A.) set to 130°C for 24 hours. We calculated water content as g H_2_O/g dry mass (g g^-1^). We repeated this procedure with seeds from the M-22 and X-22 lots. However, water content was measured immediately after the seed conditioning phase (S1 Fig). We also increased to 10 seeds per replicate, given the variation observed in measurements from 2021.

### Initial seed germination

We tested germination immediately following the two-month conditioning period by randomly selecting 500 seeds from all lots (S1 Fig) then sowing seeds on two layers of steam sterilized (40 minutes, 121°C, 117 kPa) blotter paper (Blue Steel, Anchor Paper, St. Paul, MN, U.S.A.) moistened with a 0.2% biocide solution (Plant Preservative Mixture™, PPM, Plant Cell Technologies, Washington, DC, U.S.A.) within polystyrene germination boxes (50 seeds per box, 10 boxes per lot; product number 156C, Hoffman Manufacturing, Inc., Corvallis, OR, U.S.A.). Previous research indicated that warm alternating temperatures (i.e., 35/25°C) promote wiregrass germination [46]. Therefore, we conducted germination assays on 2021 seeds at these temperatures. Subsequently, to gain a broader understanding of germination response, we selected alternating temperatures that simulated average spring or fall (28/15°C), summer (35/25°C), and winter (21/8°C) temperatures for north-central Florida for germination assays of seeds collected in 2022.

We randomly assigned two boxes per lot to incubation chambers (I-30-VL, Percival Scientific Inc., Perry, IA, U.S.A.) set to the seasonal temperatures. Chambers for all experiments were set to a 12-hour photoperiod wherein illumination coincided with maximum temperature. We continued irrigating as needed with 0.2% PPM for the 28-day experimental period. We monitored seeds daily for protrusion of shoot and radicle to at least 2 mm. The production of normal seedlings (S2 Fig) is more appropriate for understanding the likelihood of recruitment. We tested the remaining non-germinated seeds for viability using the tetrazolium staining procedure [52] and reported germination on a viable seed basis. We utilized initial germination tests as controls to assess further desiccation.

### Post-drying germination

We randomly selected 125 seeds from the 2021 lots that had been stored on the lab bench for 6 months (S1 Fig). We then divided these into samples of 100 seeds for subsequent post-drying germination testing and 25 seeds for moisture content measurements. We further divided the samples of 25 seeds into five sub-samples of five seeds each. We recorded the fresh mass of each sub-sample before placing all seeds in a glass desiccator (08-595E, Fisher Scientific, Waltham, MA, U.S.A.) above a saturated LiCl (793620, Sigma-Aldrich, St. Louis, MO, U.S.A.) solution. We prepared the LiCl solution by mixing distilled, deionized water with the salt to create a slurry [53–56]. We monitored RH and temperature within the desiccator using a data logger (Tempo Disc™ DSCTHD001, BlueMaestro, London, U.K.). Average (± SE) RH and temperature within the desiccator reached 12.7 ± 0.03% and 23.6 ± 0.12°C. We measured the fresh mass of each sub-sample every 48 hours. Seed fresh mass stabilized after one week in the desiccator. We removed all sub-samples from the desiccator after two weeks and measured the water content as described previously. We allowed the remaining 100-seed sample to adjust to RH conditions in the lab for 1 hour, then sowed these as described previously for germination testing at 35/25°C. Ultimately, we comparatively assessed the germination response between the non-LiCl desiccated seeds from the control lot described previously to the LiCl desiccated seeds used in this experiment.

We increased the number of seeds in each sub-sample from five to 10 and measured sub-sample mass every 7 days for the X-22 and M-22 seed lots. We removed seeds from the desiccator after two weeks. Average RH and temperature in the desiccator measured 11.3 ± 0.02% and 19.7 ± 0.15°C. We assessed seed water content and handled germination experiments, as mentioned before, with a few exceptions. First, we divided the seeds into six sets of 50 seeds per lot (50 seeds × 6 boxes × 2 lots). Next, to examine possible interactions between desiccation and germination temperature on germination ability, we randomly assigned two germination boxes (i.e., 100 seeds) from each population to incubators set to simulated spring or fall (28/15°C), summer (35/25°C), and winter (21/8°C) temperatures.

### Controlled aging assays

We used methods outlined by Newton et al. [57] to estimate storage longevity for seeds from all lots. We randomly selected 10 samples of 50 seeds from each of the X-21, M01-21, and M02-21 lots (n = 1,500), which had been stored in the lab for three months (S1 Fig). We placed the seeds in glass Petri dishes and then placed the dishes inside an air-tight electrical enclosure box (OPCP303013T.U, ENSTO Ltd., Porvoo, Finland) for rehydration. Relative humidity within the rehydration box remained at 47% via a non-saturated LiCl solution. We placed the rehydration box in an incubator set to 20°C without illumination then transferred the seeds to deterioration conditions after 14 days in rehydration conditions. We placed seeds within a second box maintained at 60% RH by a non-saturated LiCl solution, then transferred this deterioration box to a fan-assisted oven (Lab-Line Imperial II Incubator, Lab Line Instruments, Melrose Park, IL, U.S.A.) set to 45°C without supplemental lighting. We removed one 50 seed sample for each lot from the deterioration box after 1, 2, 5, 9, 20, 30, 50, and 75 days. Germination decreased to 0 by day 75. We sowed seeds for germination testing at 35/25°C, as described earlier.

We randomly selected 30 samples of 50 seeds from the X-22 and M-22 lots (n = 3,000) and utilized two electrical enclosure boxes for the rehydration and deterioration stages to accommodate the increased number of samples. We distributed the seeds into germination boxes after the appropriate deterioration intervals and then randomly assigned germination boxes among incubators set to three simulated seasonal temperatures (e.g., 35/25, 28/15, and 21/8°C).

### Data analysis

#### Moisture content.

We conducted one-way ANOVA to compare the moisture content of control and LiCl-dried seeds.

#### Germination.

We used non- and semi-parametric time-to-event analyses to evaluate temporal germination patterns. We coded germination events as 1 and censored events (i.e., lost, infected, or non-germinated seeds) as 0, then produced Kaplan-Meier estimates of survivor functions. We stratified survivor functions for 2021 seeds by ecotype and desiccation treatments (i.e., control vs. LiCl-dried) to test the effects of these variables on germination. Likewise, we stratified survivor functions for 2022 seeds by ecotype, desiccation treatment, and simulated seasonal temperatures. We used Kaplan-Meier estimates to generate temporal germination patterns and quantify germination parameters. The parameters included lag time (i.e., time to first germination event) in days and the time required for the failure probability (i.e., germination) to exceed 0.50. A failure probability of 0.50 represents the median (*t*_50_) germination time. We calculated germination rate as 1/*t*_50_. Estimating median germination times in cases where the failure probability did not reach 50% was not possible. Instead, we used 1/*t*_25_ as a germination rate proxy. We used the log-rank statistic to test the null hypothesis of no difference in temporal germination patterns between the desiccated and non-desiccated seeds.

Next, we modeled the likelihood of germination using Cox regression and the exact method to account for tied event times. We evaluated the proportional hazards assumption graphically and via residual analysis prior to model building then incorporated time-dependent covariates (e.g., seasonal temperature × day) into the models when violations occurred. We constructed orthogonal linear contrasts to test the null hypothesis that the coefficients for comparisons were equal.

Finally, we constructed contingency tables and then analyzed counts of normal seedlings, abnormal seedlings, and remaining viable yet non-germinated seeds using the Cochran-Mantel-Haenszel test. Normal seedlings produced true leaves and roots greater than 2 mm while abnormal seedlings were missing well-defined leaves or radicle (S2 Fig). Including these seed quality categories allowed for proportions of seedlings to be assessed on a viable seed basis. Note that a seed must be viable to germinate regardless of whether subsequent seedlings are normal or abnormal. We stratified contingency tables for seeds harvested in 2021 according to ecotype, drying treatments, and drying treatments controlling for ecotype. Contingency table stratification for 2022 harvests was by seasonal temperatures, drying treatments, and drying treatments controlling for ecotype or seasonal temperature. We conducted simple comparisons by limiting contingency table stratification in both years to the responses of normal and abnormal seedlings to assess the hypothesis of general association. We calculated Cramer’s *V* to assess effect sizes. All analyses were conducted using SAS software (v. 9.4, SAS Institute, Cary, NC, USA).

#### Controlled aging assays.

We fitted seed deterioration time courses with probit regression and estimated *K*_*i*_, *σ,* and *p*_*50*_ according to the Ellis and Roberts [6] seed viability equation, $v=K_{i}-(\frac{p}{\sigma })$ where *v* equals the probit percentage viability of the seed lot after *p* days, *K*_*i*_ represents the initial viability of the seed lot in probits, and *σ* is the standard deviation of the normal distribution of seed deaths over time (i.e., the time it takes for viability to fall by 1 probit). *p*_50_ is the product of *K*_*i*_ and *σ*; it represents the time for germination to decline to 50%. We estimated deterioration rates as the inverse of *p*_50_. We then used the Student’s t-test for one sample to compare the mean *p*_50_ values from our study to those for *Ranunculus sceleratus* (*p*_50_* *= 13.7 days) and *Brassica napus* (*p*_50 _= 65.1 days) as reported in Probert et al. [36]. Alternatively, we employed a paired t-test to evaluate mean deterioration rates for seeds from M-22 and X-22 tested across different simulated seasonal temperatures. We calculated Cohen’s d to estimate effect size. Controlled deterioration analyses were conducted using a spreadsheet (Microsoft® Excel®, v. 2406, Microsoft Inc., Redmond, WA, USA)

## Results

### Water content

**Fig 1:**
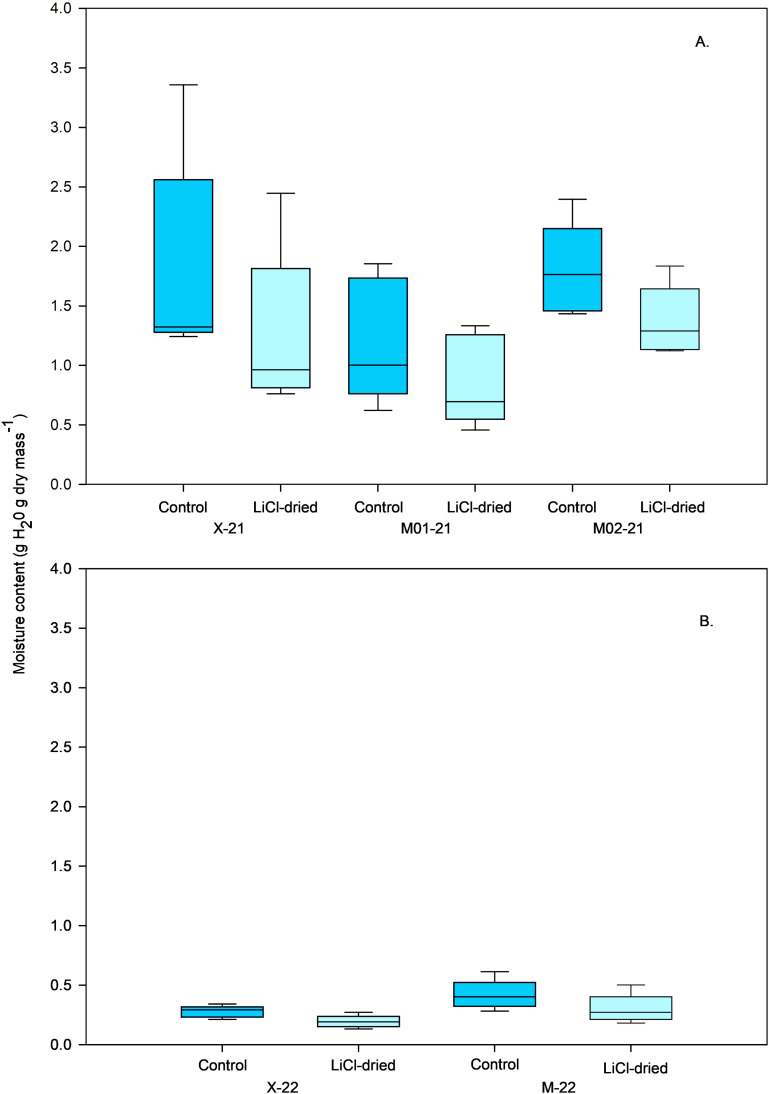
Water content of wiregrass seeds collected in: (A) 2021 from two mesic (M01-21, M02-21) and one xeric (X-21) populations and (B) 2022 from one mesic (M-22) and one xeric (X-22) population. For (A) all seeds were stored at room temperature (ca. 22°C, 50% RH) in sealed brown paper bags for six months until experiments commenced for the control (dark blue bars) and LiCl-dried (light blue bars) treatments. Seeds in the desiccation treatment were dried over a saturated LiCl solution (12% RH). For (B) all seeds were stored at room temperature until measurements were taken 2 months after harvest. Control seeds represented by dark blue bars. Seeds in the drying treatment (light blue bars) were dried over a saturated LiCl solution (11% RH).

The water content of control seeds collected from different sites in 2021 ranged from about 1.2–1.8 g g^-1^. Drying over LiCl decreased seed water content to about 0.9–1.4 g g^-1^ (Fig 1A). Seed water content for the control lots in 2022 was approximately 3–6 times lower than for seeds collected in 2021. The water content of seeds from the 2022 sites decreased further to about 0.21 g g ^−1^ following drying over LiCl (Fig 1B). There was no statistical difference in the water content of control (*F*_2,8_ = 1.40, *p* = 0.3021) or LiCl-desiccated (*F*_2,8_ = 1.40, *p* = 0.3014) seeds from the 2021 lots. Likewise, no statistical difference in water content occurred for the control (*F*_1,4 _= 5.26, *p* = 0.0835) or LiCl-desiccated (*F*_1,4 _= 3.03, *p* = 0.1576) seeds from the 2022 lots.

### Germination responses to desiccation stress

**Fig 2:**
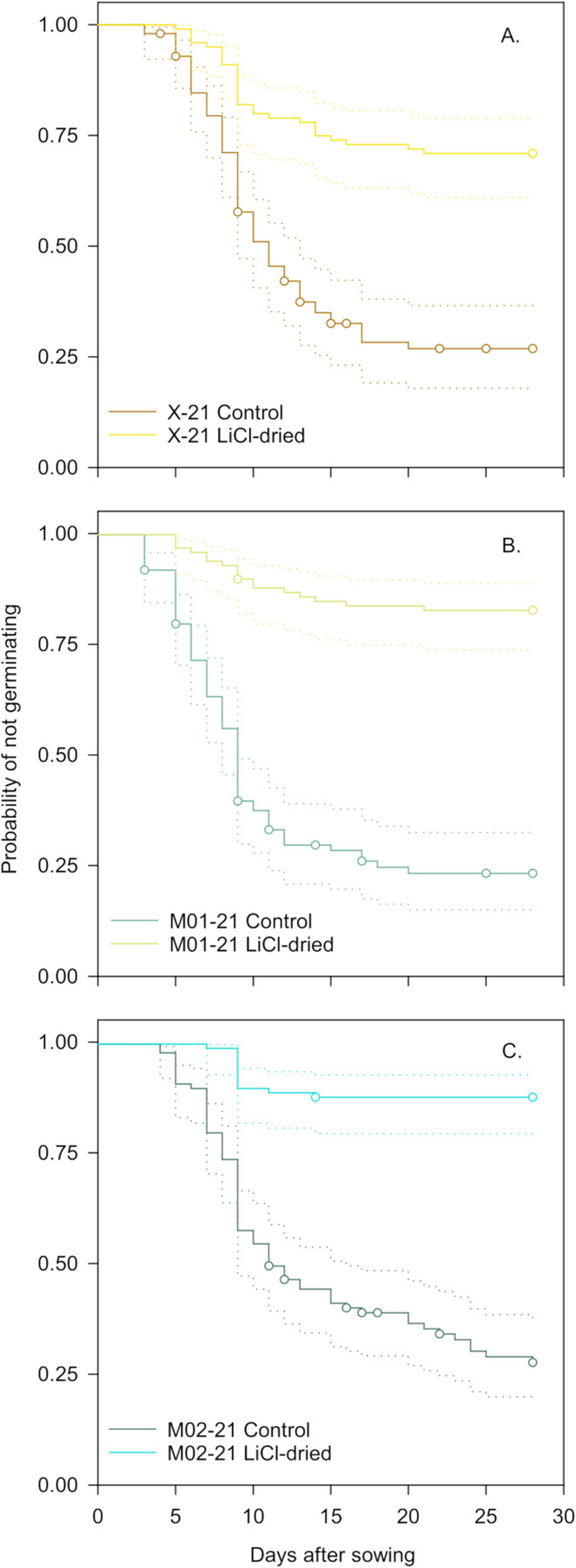
Kaplan-Meier estimates of survivor functions for control and LiCl-dried wiregrass seeds germinating under 35/25°C. All seeds were stored at room temperature (ca. 22°C, 50% RH) in sealed brown paper bags until experiments commenced after 2 and 6 months of laboratory storage for the control and dried treatments, respectively. Seeds in the desiccation treatment were dried over a LiCl saturated salt solution (12% RH) prior to germination. Dotted lines indicate 95% confidence limits. Circles represent censored observations.

Temporal patterns indicated that germination was relatively rapid for control (i.e., non-LiCl dried) seeds collected in 2021. Germination lag for these seeds was 3–4 days, and the germination rate based on 1/*t*_50_ ranged between 0.09–0.11. Final germination ranged from about 87–90% (Fig 2A; Table 1). However, LiCl-dried seeds displayed comparatively reduced germination at 35/25°C across all sites. For example, germination percentage for LiCl-dried seeds was about 2.3–5.2 times lower than control seeds. Germination lag was 1.3–2.3 times longer in desiccated seeds than control seeds. Failure probabilities surpassed 0.25 for only one LiCl-dried seed lot in 2021. Therefore, we used 1/*t*_25_ as a germination rate proxy. Germination rates were 1.9–2.4 times faster for control compared to the LiCl-dried seeds harvested from a xeric site (Fig 2; Table 1). The log-rank test provided sufficient statistical evidence (Log-rank $\begin{array}{c} \chi_{\text{5}}^{\text{2}} \end{array}$ = 194.65, *p* < 0.0001) to reject the null hypothesis of no differences in temporal germination patterns when stratifying by ecotype and desiccation treatment.

**Table 1:** Germination parameters for *Aristida beyrichiana* seeds collected in 2021 or 2022 from xeric (X) or mesic (M) habitats. Seeds were either dried over saturated LiCl solutions or not exposed to further desiccant-based drying.

Temperature	Lot	Treatment	Germination (%)^z^	Lag (d)	*t*_25_	*t*_50_	1/*t*_25_	1/*t*_50_
35/25°C	X-21	Control	89.3	3	8 [7,9]	11 [9,13]	0.13	0.09
		LiCl-dried	39.7	5	14.5 [9,-]	- ^y^	0.07	–
	M01-21	Control	90.1	3	6 [5,7]	9 [8,9]	0.17	0.11
		LiCl-dried	22.4	5	-^y^	–	–	–
	M02-21	Control	87.3	4	8 [7,9]	11.5 [9,17]	0.13	0.09
		LiCl-dried	16.9	7	–	–	–	–
								
35/25°C	X-22	Control	84.9	3	6 [6,7]	10 [8,14]	0.17	0.10
		LiCl-dried	74.4	3	7 [6,8]	10 [9,13]	0.14	0.10
	M-22	Control	88.0	3	6 [5,7]	9 [7,10]	0.17	0.11
		LiCl-dried	81.7	2	6 [5,7]	9 [8,11]	0.17	0.11
								
28/15°C	X-22	Control	79.3	6	9 [8,10]	12 [11,14]	0.11	0.08
		LiCl-dried	71.6	6	9 [8,12]	16 [13,20]	0.11	0.06
	M-22	Control	86.6	5	9 [8,10]	12 [11,13]	0.11	0.08
		LiCl-dried	80.2	6	9 [7,10]	13 [11,15]	0.11	0.08
								
21/8°C	X-22	Control	66.7	10	18 [15, 20]	25 [21,-]	0.06	0.04
		LiCl-dried	56.6	10	18 [17,22]	–	0.06	–
	M-22	Control	72.3	12	16 [15,18]	22 [19,26]	0.06	0.04
		LiCl-dried	60.0	12	17 [16,19]	27 [21,-]	0.06	0.04

z Germination percentage determined on a viable seed basis.

y The Kaplan–Meier estimator for these data did not reach a failure probability <0.25 or 0.50.

**Fig 3:**
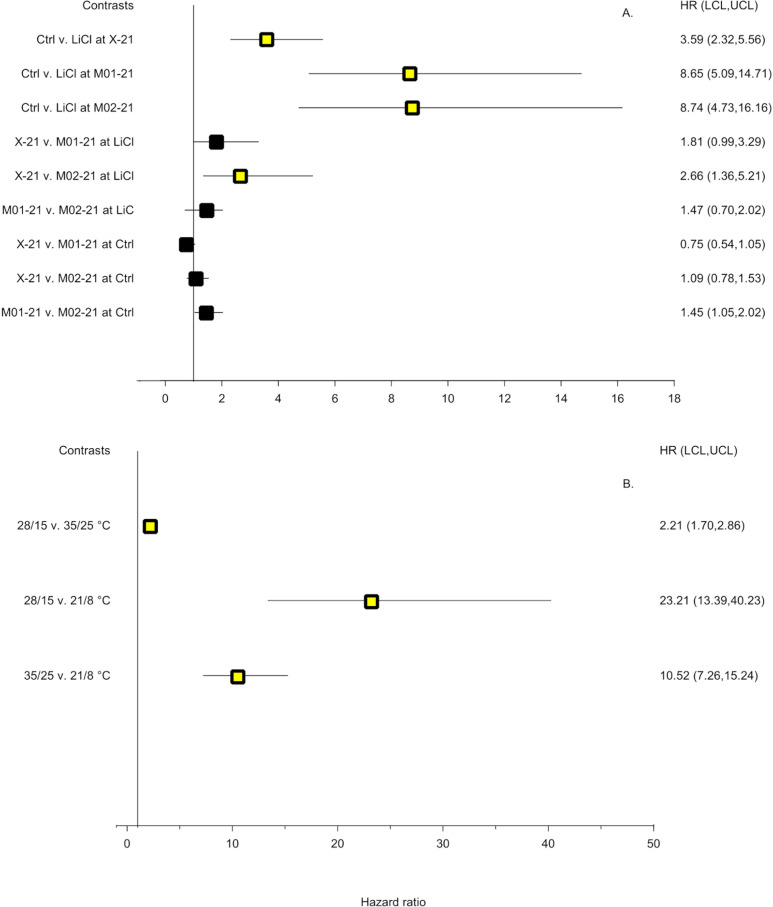
Forest plots depicting linear orthogonal contrasts of wiregrass germination for seeds collected in (A) 2021 and (B) 2022. Seeds were dried over saturated LiCl solutions (LiCl) or remained undried (Ctrl) prior to germination. In (A) X-21, M01-21, and M02-21 represent seed lots collected from xeric (X) or mesic (M) habitats during 2021. In (B) 28/15, 35/25, and 21/8°C represent simulated temperatures during the spring or fall, summer, and winter, respectively, Squares and horizontal lines represent values for hazard ratios and confidence limits, respectively. The vertical black line represents the reference line equal to 1.00 for determination of statistical significance. Confidence limits bracketing the reference line denote a non-significant comparison. Yellow and black squares also denote significant and non-significant hazard ratios, respectively. Squares to the right of the reference line denote that the first factor in a comparison displayed a greater likelihood of germination than the second factor. Squares to the left of the reference line denote that the second factor in the comparison displayed a higher likelihood of germination. Taking the reciprocal 1/HR of values less than 1 provides a comparable hazard ratio.

Cox regression detected a statistically significant ecotype × desiccation treatment interaction ($\begin{array}{c} \chi_{\text{2}}^{\text{2}}\text{ = 6.74, p=0.01};\; \end{array}$ S2 Table). Subsequent orthogonal contrasts (Fig 3A) indicated that the likelihood of germination was about 3.6–8.7 times greater for control seeds than LiCl-dried seeds. The likelihood of germination for most inter-ecotype comparisons within LiCl-dried or control treatments ranged between about 1.1–1.8 and were not statistically significant. The only exception occurred when comparing xeric and mesic seeds dried with LiCl. Here, the likelihood of germination was about 2.7 times greater for xeric compared to mesic seeds (Fig 3A).

**Fig 4:**
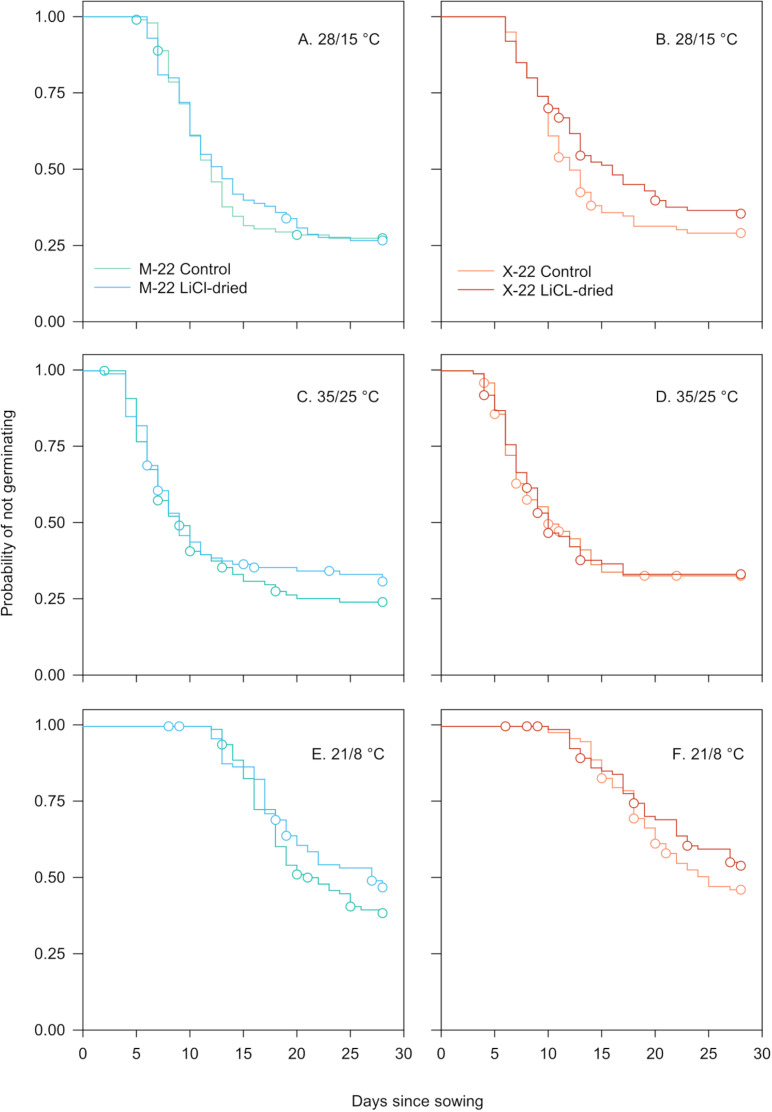
Kaplan-Meier estimates of survivor functions for control and LiCl desiccated wiregrass seeds from two sources (M-JUL-22 and X-JUL-22) germinated under simulated seasonal temperatures representing (A–B) spring or fall (28/15°C), (C–D) summer (35/25°C), and (E–F) winter (21/8°C) in north-central Florida. Seeds in the desiccation treatment were dried to 11% RH via LiCl saturated salt solution prior to germination. Circles represent censored observations. Ninety-five percent confidence limits omitted for clarity.

Temperature rather than ecotypic or desiccation-based differences in temporal germination patterns were evident for seeds collected in 2022. For instance, germination lag ranged from 2–6 days for seeds treated with spring or fall (28/15°C) or summer (35/25°C) temperatures. However, germination lag was 1.7–6.0 times longer for seeds exposed to winter (21/8°C) conditions. Likewise, germination was 1.5–2.8 times slower under winter compared to remaining seasonal temperatures (Fig 4; Table 1). Similar patterns emerged for germination percentage. For example, germination ranged from about 57–72% for seeds exposed to winter temperatures (21/8°C) but increased to nearly 72–88% at remaining simulated seasonal temperatures. However, final germination percentage was only about 1.1–1.2 times lower in LiCl-dried seeds compared to control seed (Table 1). The log-rank test provided adequate statistical evidence (Log-rank $\begin{array}{c} \chi_{\text{5}}^{\text{2}} \end{array}$ = 194.65, *p* < 0.0001) to reject the null hypothesis of no differences in temporal germination patterns when stratifying by ecotype, simulated seasonal temperature, and desiccation treatment.

Cox regression indicated that seasonal temperature was the only statistically significant ($\chi_{2}^{2}$ = 101.71, *p* < 0.0001) effect on germination for seeds collected in 2022 (S2 Table). Ensuing orthogonal contrasts specified that the effect of temperature was largest when comparing germination under spring or fall (28/15°C) and (35/25°C) summer temperatures to winter temperatures (21/8°C). For example, the likelihood of germination was about 11–23 times greater for seeds incubated at spring or fall and summer temperatures compared to winter temperatures. A more muted yet statistically significant response was evident when comparing germination at spring or fall to summer conditions (Fig 3B).

The absolute differences in percentages between normal and abnormal seedlings were small and ranged from about 1–11 percentage points (% pts) when grouped by ecotype for 2021 seeds. Xeric seeds produced more normal seedlings than abnormal seedlings. However, mesic ecotype seeds produced slightly more abnormal than normal seedlings (S3A Fig). The association between ecotype and seedling status was negligible and not statistically significant (Table 2). A more prominent effect of drying treatment on seedling status was evident for seeds harvested in 2021. Normal seedling production from control seeds was about 56% pts higher than abnormal seedlings. However, LiCl-dried seeds produced about 24% more abnormal than normal seeds (S3B Fig). Drying treatments were moderately associated with seedling status (Table 2). Evaluation of the association between drying treatments and seedling status when controlling for ecotype revealed more detailed differences in normal and abnormal seedling production. Here, control seeds produced more normal seedlings than LiCl-dried seeds (range: about 37–41% pts). Yet, the number of abnormal seedlings produced by LiCl-dried seeds was always greater than control seeds by a difference of 37–43% pts (S3C–S3E Fig). Simple comparisons revealed a moderate and statistically significant association between drying treatments and seedling status when controlling for ecotype (Table 2).

**Table 2:** Statistical results for analysis of associations between ecotype (xeric or mesic sites), drying treatments (control, LiCl-dried), simulated seasonal germination temperatures (spring or fall = 28/15°C, summer = 35/25°C, winter = 21/8°C) and counts of normal or abnormal wiregrass seedlings and remaining non-germinated yet viable seeds following germination tests. Cramér’s *V* represents a measure of effect magnitude. Degrees of freedom (df) for Cramer’s *V* are calculated as the minimum of the columns and rows minus 1 (min[c-1, r-1]) in a contingency table. Contingency tables in for the above analyses were either 3 × 2 or 2 × 2. Therefore, df for calculations of *V* always equaled one. Cohen (1998) offers general guidelines for interpretation of Cramér’s *V* when no discipline specific criteria exist. The guidelines for interpretation of *V* when df = 1 are: 0 < 0.10, negligible; 0.10 < 0.30, small; 0.30 < 0.50, medium; ≥ 0.50, large.

Variable	*χ*^2^ [df, *N*]	*p*	Cramér’s *V*	95% CI	Interpretation
*2021*					
Ecotype × seedling status (normal, abnormal)	3.08 [4, 336]	0.5450	0.07	0.00, 0.14	negligible
Drying treatment × seedling status	48.43 [1, 336]	<0.0001	0.37	0.28, 0.47	medium
*Simple comparisons*					
Drying treatment × seedling status controlling for ecotype (X-21)	16.69 [1, 95]	<0.0001	0.42	0.25, 0.57	medium
Drying treatment × seedling status controlling for ecotype (M01-21)	16.07 [1, 98]	<0.0001	0.41	0.21, 0.57	medium
Drying treatment × seedling status controlling for ecotype (M02-21)	12.89 [1, 104]	0.0003	0.35	0.17, 0.51	medium
*2022*					
Ecotype × seedling status	0.01 [2, 451]	0.9948	0.00	0.01, 0.13	negligible
Simulated seasonal temperature × seedling status	11.94 [4, 451]	0.0178	0.12	0.07, 0.18	small
Drying treatment × seedling status	9.95 [2, 451]	0.0069	0.15	0.07, 0.25	small
*Simple comparisons*					
Seasonal temperature × seedling status controlling for, mesic, control	1.00 [2, 73]	0.6074	0.12	0.04, 0.36	small
Seasonal temperature × seedling status controlling for, mesic, LiCl-dried	1.46 [2, 79]	0.4810	0.14	0.04, 0.38	small
Seasonal temperature × seedling status controlling for, xeric, control	3.47 [2, 93]	0.1760	0.19	0.07, 0.42	small
Seasonal temperature × seedling status controlling for, xeric, LiCl-dried	0.80 [2, 90]	0.6701	0.09	0.03, 0.32	negligible
Drying treatment × seedling status controlling for mesic, spring	3.47 [1, 43]	0.0627	0.28	0.03, 0.57	small
Drying treatment × seedling status controlling for mesic, summer	0.64 [1, 51]	0.4239	0.11	0.01, 0.37	small
Drying treatment × seedling status controlling for mesic, winter	1.24 [1, 58]	0.2655	0.15	0.00, 0.41	small
Drying treatment × seedling status controlling for xeric, spring	0.19 [1, 49]	0.6660	0.06	0.01, 0.35	negligible
Drying treatment × seedling status controlling for xeric, summer	2.38 [1, 59]	0.1232	0.20	0.01, 0.45	small
Drying treatment × seedling status controlling for xeric, winter	0.30 [1, 75]	0.5850	0.06	0.00, 0.30	negligible

Seeds collected in 2022 displayed considerably different patterns in seedling status than those collected in 2021. For example, stratifying by ecotype, seasonal temperature, or drying treatments revealed that normal seedlings surpassed abnormal seedlings by about 7–30% pts (S4A–S4C Fig). However, the association between these variables and seedling status was negligible to small and only statistically significant for drying treatments (Table 2). Similarly, normal seedling production was about 4–29% pts greater for control compared to LiCl-dried seeds when stratifying by seasonal temperature, drying treatment, and ecotype. In all but one instance, LiCl-dried seeds produced 4–16% pts more abnormal seedlings than control seeds (S4D–S4I Fig). The difference in abnormal seed production for the exception was < 1% (S4G Fig). Simple comparisons indicated that the association between 1) seasonal temperatures and seedling status controlling for ecotype and drying treatment or 2) drying treatments and seedling status controlling for ecotypes and seasonal temperatures were negligible to small. None of these comparisons were statistically significant (Table 2).

### Controlled aging assays

**Fig 5:**
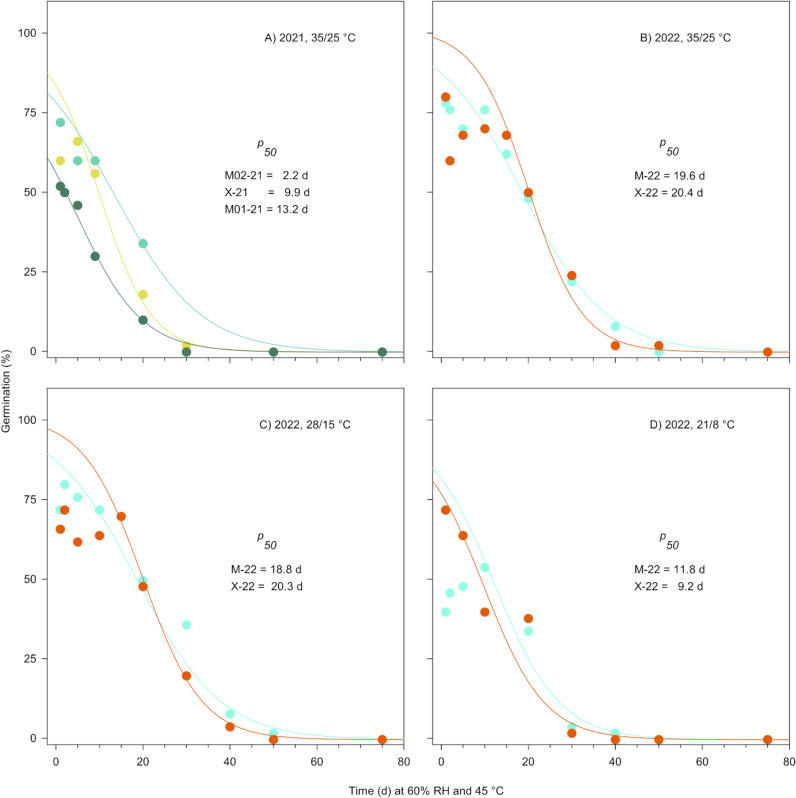
Deterioration curves for wiregrass seeds harvested in (A) 2021 from mesic (M01-21, M02-21) and xeric (X-21) sites then incubated at simulated summer (35/25°C) temperatures after controlled deterioration treatments or (B) 2022 from mesic (M-22) or xeric (X-22) sites then incubated at simulated summer (35/25°C), spring or fall (28/15°C), and winter (21/8°C) temperatures following controlled deterioration treatments. The *p*_50_ values denote the time in days for germination to decline to 50%.

The mean *p*_50_ value for 2021 seeds reached 8.4 days (95% CI [3.2, 13.9]; Fig 5A), and the effect of ecotype on *p*50 values was not statistically significant (*t*_2_ = 2.56, *p* = 0.1244). The magnitude of seed deterioration effects induced by the controlled aging assays on germination differences between wiregrass seeds and *R. scleratus* (d = 0.87, 95% CI [0.57, 2.20], *t*_2_ = −1.51, *p* = 0.2696) or *B. napus* (d = 9.70, 95% CI [1.44, 18.70], (*t*_2_ = −16.80, *p* = 0.0035) were large as denoted by Cohen’s d. However, these effect sizes should be interpreted cautiously, given the wide confidence intervals and relatively small sample sizes. Nonetheless, insufficient statistical evidence existed to reject the null hypothesis of no difference in mean *p*_50_ values for wiregrass and short-lived *R*. *scleratus* seeds (*p*_50_ = 13.7 days). Meanwhile, the difference in *p*_50_ between wiregrass and long-lived *B. napus* seeds (*p*_50_ = 65.1 days) was deemed statistically significant. Furthermore, no clear ecotypic patterning in deterioration was evident for seeds collected in 2021. For example, the deterioration rate (i.e., 1/*p*_50_) for seeds from the xeric habitat was bracketed by deterioration rates for seeds from the mesic habitat (Fig 5A).

Seeds collected in 2022 generally displayed *p*_50_ values about 5–18 days greater than *p*_50_ values for 2021 seeds. Moreover, *p*_50_ values for seeds collected in 2022 and then exposed to simulated winter (21/8°C) temperatures decreased by about 1.7–2.2 times compared to warmer seasonal temperatures (Fig 5B–5D). Regardless, small and non-statistically significant effects of ecotype on post-deterioration germination were evident (d = 0.22, 95% CI [0.00, 0.44], *t*_2_ = −0.68, *p* = 0.5717). Small to medium, yet non-statistically significant, effects were apparent for differences in *p*_50_ values between mesic (d = 0.71, 95% CI [−0.65, 1.95], *t*_2_ = 1.22, *p* = 0.3455) or xeric (d = 0.45, 95% CI [−0.80, 1.61], *t*_2_ = 0.78, *p* = 0.5165) seeds when compared to *R*. *scleratus*. However, large effects for differences in *p*_50_ values between *B*. *napus* and both ecotypes were evident (mesic: d = 11.27, 95% CI [1.70, 21.70], *t*_2_ = −19.52, p = 0.0026; xeric: d = 7.50, 95% CI [1.05, 14.48], *t*_2_ = −12.99, p = 0.0059).

## Discussion

Seeds with unusual storage physiology pose challenges to seed sourcing strategies for restoration practice. Identification of species with such traits is therefore crucial. Here, we examined seed water content, germination, desiccation tolerance, and relative ex situ seed longevity in relation to ecotypic variation and germination temperature of wiregrass, a dominant, foundation species of high restoration value for pine ecosystems throughout the NACP. We found that wiregrass seeds, regardless of ecotype, germinate preferentially at warm temperatures and display considerable desiccation tolerance. However, we estimate seeds from both xeric and mesic habitats to be short-lived during ex situ storage.

The relatively high moisture content (range 0.9–1.8 g g-1) for all seeds harvested in 2021 is insufficient for germination but corresponds to hydration levels that allow for respiration and catabolic reactions, which may manifest as stress metabolism [23,25]. A common yet unfortunate misconception is that indoor areas provide satisfactory conditions for extended seed storage in open containers. However, guidelines for indoor human comfort suggest temperatures between 20–27°C with 30–60% RH [58], which is well above the temperature and RH guidelines for seed storage [12–15]. Additionally, the ambient conditions within a climate-controlled room can vary considerably depending on various factors [58]. This variation may have greater impacts on the viability maintenance of short-lived than long-lived seeds [23,28].

Thus, the extended laboratory storage of 2021 seeds might have hastened deterioration and influenced subsequent results. Cells forming the radicle are often primary sites of accumulated aging damage thereby negatively impacting the completion of germination and subsequent seedling establishment. But cells in other organs are also susceptible [22,59–62]. We suspect that aging damage in wiregrass seeds occurs throughout the embryo given the proportion of abnormal seedlings without further radicle or cotyledon development (S2–S3 Figs). The physiological basis for such damage in wiregrass seeds is currently unknown. However, previous studies indicate that aging damage may be the result of an array of biochemical and physical dysfunctions that are not resolved upon rehydration. These include factors such as hydrolytic, oxidative, and peroxidative reactions that degrade lipids, proteins, and nucleic acids; physical damage to DNA and RNA that reduce molecule integrity; and membrane phase changes [9,16,25,63–69].

Drying seeds that are already deteriorating, such as those collected in 2021, provides no benefit, as evidenced by subsequent diminished germination responses and production of higher proportions of abnormal seedlings for LiCl-dried seeds. There is evidence to suggest that imbibition damage can occur in dry seeds that are insufficiently pre-humidified prior to exposure to free water during sowing. In this instance, water rapidly enters embryonic cells and perturbs reformation of cell membrane bilayer integrity [25]. We would expect that LiCl-dried seeds from 2022 would experience large declines in germination relative to controls if imbibitional damage was present. However, we pre-humidified seeds from 2021 and 2022 in the lab for one hour prior to sowing and did not observe large scale decreases in germination for LiCl-dried seeds from 2022 (Table 1). Therefore, the decrease in germination for 2021 seeds following drying over LiCl appears to be related to aging stress from previous storage which may be compounded by desiccation stress.

Considerable reductions in germination capacity coupled with abnormal seedling growth (Table 1; Fig 2; S3 Fig) are common symptoms of accumulated damage due to one or more stressors and indicative of seed vigor losses preceding viability declines [70–72]. The challenges of establishing seedlings from viable seeds in direct-seeded habitat restoration programs are well known [73,74]. It is also well known that the seed to seedling transition represents the most stress-susceptible plant life stage [75]. An implicit assumption is that, despite existing stressors, seedlings will arise from viable seeds. However, even if viable initially, subsequently compromised seeds will produce abnormal seedlings with an extremely low probability of survival in the field. Second, the use of compromised seeds will likely increase costs when attempting to realize restoration outcomes. Third, proper post-harvest drying is crucial for wiregrass storage and subsequent seedling production.

The water content of 2022 seeds aligned more closely with species expressing some degree of desiccation tolerance. Although water content is not the absolute predictor of desiccation tolerance, the LiCl-induced moisture levels tolerated by wiregrass seeds in this study are below the lower limits (e.g., −15 MPa) defining the recalcitrant storage physiology [23,25]. The survival of wiregrass seeds to LiCl-induced water contents also coincides with mature seed water content of other wild grasses displaying desiccation tolerance and considered to express intermediate storage physiology [36,51,76,77].

It is interesting to note that a small proportion of non-LiCl dried 2022 seeds still produced abnormal seedlings. This suggests that sub-populations of wiregrass seeds may have variable desiccation tolerance phenotypes. Alternatively, despite timing our seed harvesting to coincide with the natural seed shedding period, it is possible that some seeds on a given panicle may not have reached a developmental stage where desiccation tolerance is fully expressed. A single wiregrass plant produces thousands of seeds. There is no guarantee that all reproductive mechanisms on wiregrass plants are synchronized. This can lead to sub-populations of seeds at marginally different maturity levels expressing variable desiccation tolerance. Nonetheless, and in conjunction with the improved germination responses and reduced proportion of abnormal seedlings following LiCl drying for 2022 seeds, we propose that most wiregrass seeds harvested at the point of natural shedding tolerate desiccation levels necessary for storage. This is crucial as seeds that tolerate storage relevant drying tend to also benefit by cold temperatures that extend viability during storage [6,7]. Further studies on wiregrass investigating changes in seed-water relations and cellular volume during development, seed chemical composition, and relationships between viability maintenance following factorial combinations of low water contents (e.g., 3–12%), storage temperatures (e.g., −20 to 10°C), and storage durations (e.g., 3–12 months) could pinpoint seed storage physiology [12–16,22,27]. This could help develop appropriate storage methods for wiregrass and other species of high restoration value.

Curiously, wiregrass seeds are likely short-lived in storage despite expressing desiccation tolerance. Seeds presenting *p*_*50*_ less than or equal to 25 days are considered short-lived [32,37]. This highlights the importance of appropriate post-harvest handling and storage conditions, especially for seeds that may display intermediate storage physiology. For example, long-term preservation of intermediate *Zizania palustris* (Poaceae) seeds was considered feasible with proper seed moisture and temperature management [77]. It also emphasizes the inadequacy of wiregrass seed storage in non-climate-controlled structures. Exposure to highly variable temperature and relative humidity conditions in these types of structures accelerates deterioration, especially in the persistently warm, humid conditions encountered throughout the NACP [53]. Moreover, although all *p*_*50*_ values for wiregrass seeds were below 25 days, it is worth noting that values can be greatly affected by the chosen germination temperatures (Fig 5). Hence, more complete analyses of germination under various thermal scenarios are essential for wild species when estimating potential storage longevity.

Although longevity in the soil is not necessarily linked to storage longevity, the longevity of wiregrass seeds in either location seems limited. For example, various studies in the NACP found no evidence that wiregrass forms transient or persistent soil seed banks [78–80]. Likewise, Kaeser and Kirkman [81], using wiregrass seeds with 95% initial viability, found 0% viable seeds after burial for one year. Alternatively, Mulligan and Kirkman [42] and others (J. Fill and R. Crandall 2024, personal communication) detected wiregrass recruitment up to two years after initial seed shedding or sowing and attributed this to formation of a short-term persistent seed bank [10]. These inconsistent observations are compatible given the genetic underpinnings of seed longevity and the influence of variable environmental conditions on expression of this plastic trait [11]. Nonetheless, the extent of correlations between soil and storage longevity of wiregrass seeds deserves further investigation given the importance to restoration strategies.

The innate ability of seeds to tolerate post-harvest desiccation and aging stress while also expressing longevity during storage is programmed during seed development. Several authors report that environmental conditions sensed by the mother plant and developing seeds can modulate the expression of these traits [82,83]. Accordingly, habitat of origin influences inter-specific differences in seed storage physiology [26,30,31]. Yet, factors driving intra-specific variability associated with seed desiccation tolerance and potential storage longevity across ecotypes are not well understood.

Results from wiregrass harvested in 2021 give the impression that seeds from xeric environments may be better adapted to post-harvest drying than seeds from mesic habitats. However, 2021 seeds were already further along in terms of deterioration. Alternatively, the negligible ecotypic effects observed for 2022 seeds cast doubt on the idea that habitat of origin serves as a convincing predictor of seed desiccation tolerance at the intra-specific level. Furthermore, we did not find convincing evidence that habitat of origin influences wiregrass deterioration to an appreciable extent for 2021 or 2022 seeds. Instead, species-level taxonomy may be a better predictor of wiregrass seed desiccation tolerance, and perhaps relative ex situ longevity, than habitat.

Finally, while wiregrass germination appears possible during any season, warmer temperatures experienced during spring or fall, and summer have the largest positive effects on germination response compared to germination under winter temperatures. The promotive influence of warmer temperatures makes sense given the seed dispersal phenology of this species. For example, seed shedding occurs at the onset of winter. Winter in north-central Florida is relatively mild but dry. However, periods of lethal freezing temperatures commonly occur. Shifting most germination events to warmer, wetter periods can increase the probability of avoiding temperatures or low soil moisture that could otherwise kill seedlings. Also, evolving strategies that permit germination to occur, albeit at comparatively reduced levels and rates of total normal seedling production, when temperatures are lower can provide opportunities for increased seedling establishment during milder, wetter winters when the risk of freezing events is lower. Such broad, permissive germination temperatures enhance the abundance of individuals and influence population or community structure [ 10,84].

## Conclusion

Seed storage represents an increasingly important aspect of ecological restoration programs globally. Having a seed inventory stored under conditions that maintain viability and vigor from one season to the next or longer can benefit restoration practice by providing a ready supply of germplasm to conduct plant establishment activities. However, focusing attention on adequate pre-storage seed drying and moisture management during storage are crucial. This takes on greater relevance, even within indoor air-conditioned locations, for seeds estimated to express reduced storage longevity. The suggested conditions for indoor human comfort are outside the levels suggested for seed storage. Maintaining seeds at hydration levels, like those experienced by wiregrass, that induce deterioration create uncomfortable conditions for viability maintenance of short-lived seeds. Therefore, optimizing actions that limit deterioration, like appropriate collection timing and proper packaging to prevent wide variation in water content as demonstrated for 2021 seeds should occur soon after harvest for species with atypical seed storage responses. Moreover, expression of unusual storage physiology does not necessarily equate with sensitivity to desiccation stress that could limit seed viability in the field. Instead, matching seeding activities to periods that reflect appropriate thermal and soil moisture conditions for germination can maximize the production of normal seedlings. This may mean taking advantage of fall, spring, or summer periods for wiregrass seeding activities in the field. Or, simulating those conditions in propagation facilities. Additionally, any seeding activities should account for the high number of empty seeds produced by wiregrass. Finally, consideration of estimated storage longevity and thermal germination cues apply regardless of adult plant ecotypic adaptation.

## Supporting information

S1 Fig Two-year experimental workflow. Experimental workflow for wiregrass seeds harvested in 2021 and 2022.
(PDF)

S2 Fig *Aristida stricta* (wiregrass) seedlings. Images of (A) abnormal wiregrass seedlings missing a well-defined cotyledon and leaves or radicle and roots and (B) normal seedling with true leaves and roots. Scale bars in A and B = 1 cm.
(JPG)

S3 Fig Normal and abnormal seedling production for 2021 harvest. Relative frequency of normal (green bars) and abnormal (gray bars) wiregrass seedlings grouped by (A) ecotype of seed collections (xeric = X-21; mesic = M01-21, M02-21) and (B) drying treatments (control or LiCl-dried) for seeds collected in 2021. Panels C-D show seedling frequencies grouped by drying treatments and seedling status controlling for ecotypes. In (C) brown and yellow bars denote control and LiCL-dried treatments, respectively. Accordingly, in (D) light green bars = control and chartreuse bars = LiCl-dried seeds, and in (E) dark green bars = control and aqua bars = LiCl-dried seeds. Seedling frequencies are reported on a viable seed basis.
(JPG)

S4 Fig Normal and abnormal seedling production for 2022 harvest. Relative frequency of normal (green bars) and abnormal (gray bars) wiregrass seedlings grouped by (A) ecotype of seed collections (xeric, mesic), (B) simulated seasonal germination temperature (spring = 28/15°C, summer = 35/25°C, winter = 21/8°C), and (C) drying treatments (control or LiCl-dried) for seeds collected in 2022. Panels D-I show frequencies grouped by drying treatments, ecotypes, and simulated seasonal temperatures. In D-F light green and blue bars denote control and LiCL-dried treatments, respectively. In G-I orange and red bars denote control and LiCl-dried treatments, respectively. Seedling frequencies are reported on a viable seed basis.
(JPG)

S1 Table Wiregrass seed collection information. Wiregrass seed collection sites, with names used in the manuscript, and type of habitat where plants occurred. Sites M01-21 and M02-21 represent different burn units separated by about 2 km.
(PDF)

S2 Table Cox regression results. Final Cox regression models for germination of wiregrass seeds collected in 2021 and 2022. The model for germination in 2022 contains the time-dependent covariate Seasonal temperature × Days to account for non-proportionality of the germination response to seasonal temperatures.
(PDF)
