# Supplementary material for: Desiccation tolerant yet short-lived seeds: A conundrum for post-harvest handling of a high restoration value bunchgrass?
Source: PLoS One. 2025 Jun 20;20(6):e0326596. doi: 10.1371/journal.pone.0326596 (PMC12180627; doi:10.1371/journal.pone.0326596)
Supplement: S1 Fig — Experimental workflow for wiregrass seeds harvested in 2021 and 2022. (PDF) [file pone.0326596.s001.pdf]

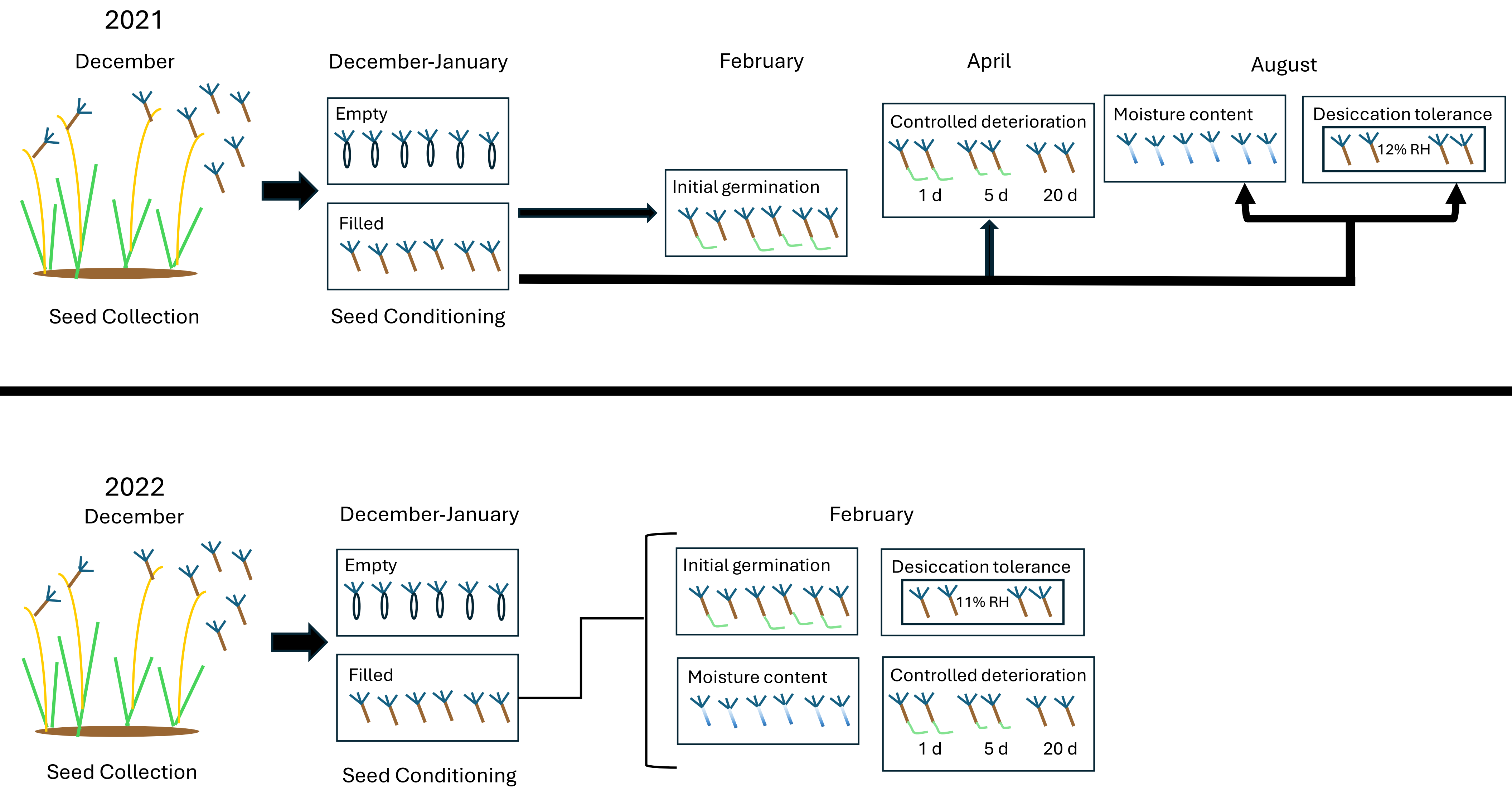
2021
December
December-January
February
April
August
Empty
Filled
Initial germination
Controlled deterioration
1 d
5 d
20 d
Moisture content
Desiccation tolerance
12% RH
Seed Collection
Seed Conditioning
2022
December
December-January
February
Empty
Filled
Initial germination
Desiccation tolerance
11% RH
Moisture content
Controlled deterioration
1 d
5 d
20 d
Seed Collection
Seed Conditioning
