# Supplementary figures and images for: Desiccation tolerant yet short-lived seeds: A conundrum for post-harvest handling of a high restoration value bunchgrass?
Source: PLoS One. 2025 Jun 20;20(6):e0326596. doi: 10.1371/journal.pone.0326596 (PMC12180627; doi:10.1371/journal.pone.0326596)

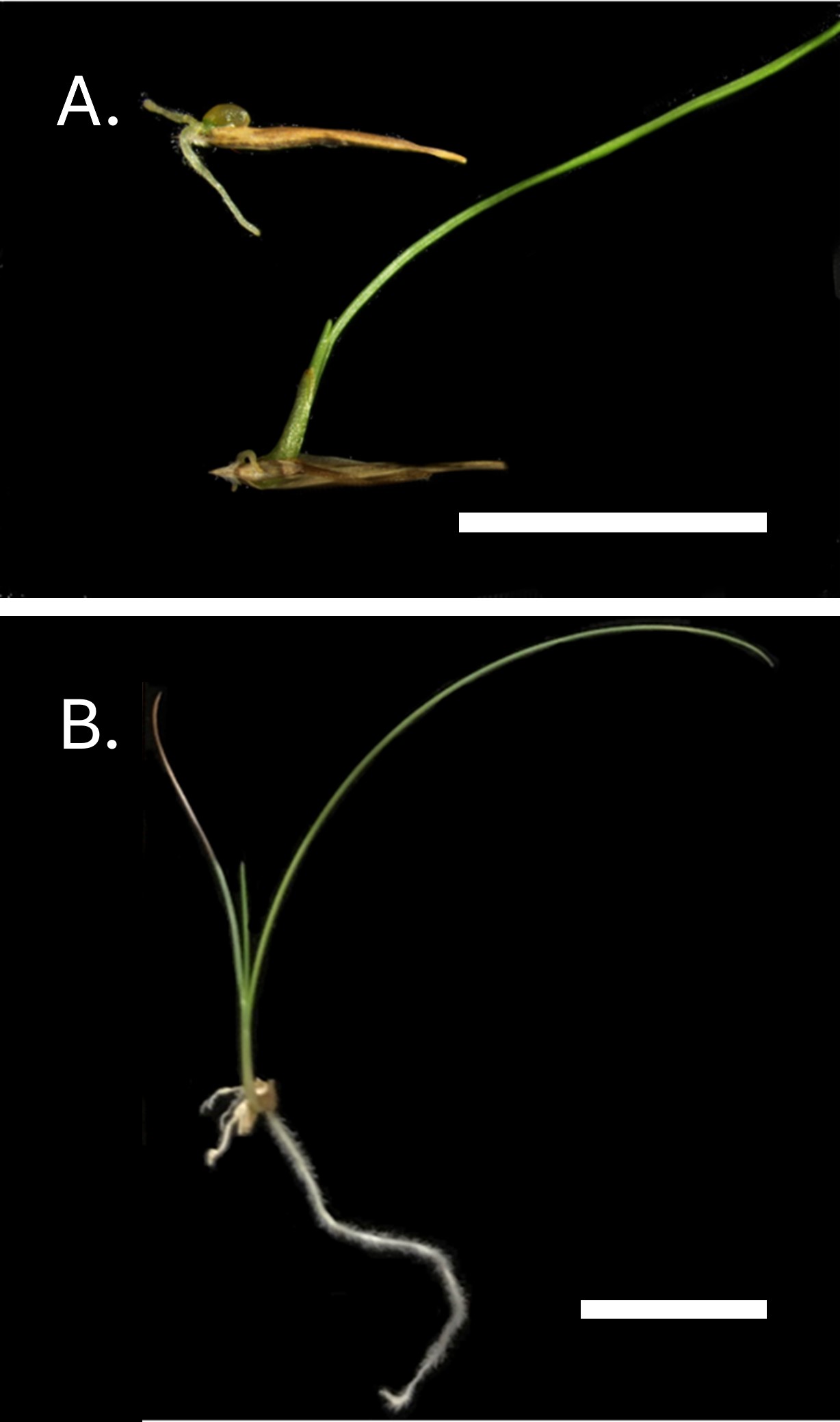

Supplement: S2 Fig — Images of (A) abnormal wiregrass seedlings missing a well-defined cotyledon and leaves or radicle and roots and (B) normal seedling with true leaves and roots. Scale bars in A and B = 1 cm. (JPG) [file pone.0326596.s002.jpg]

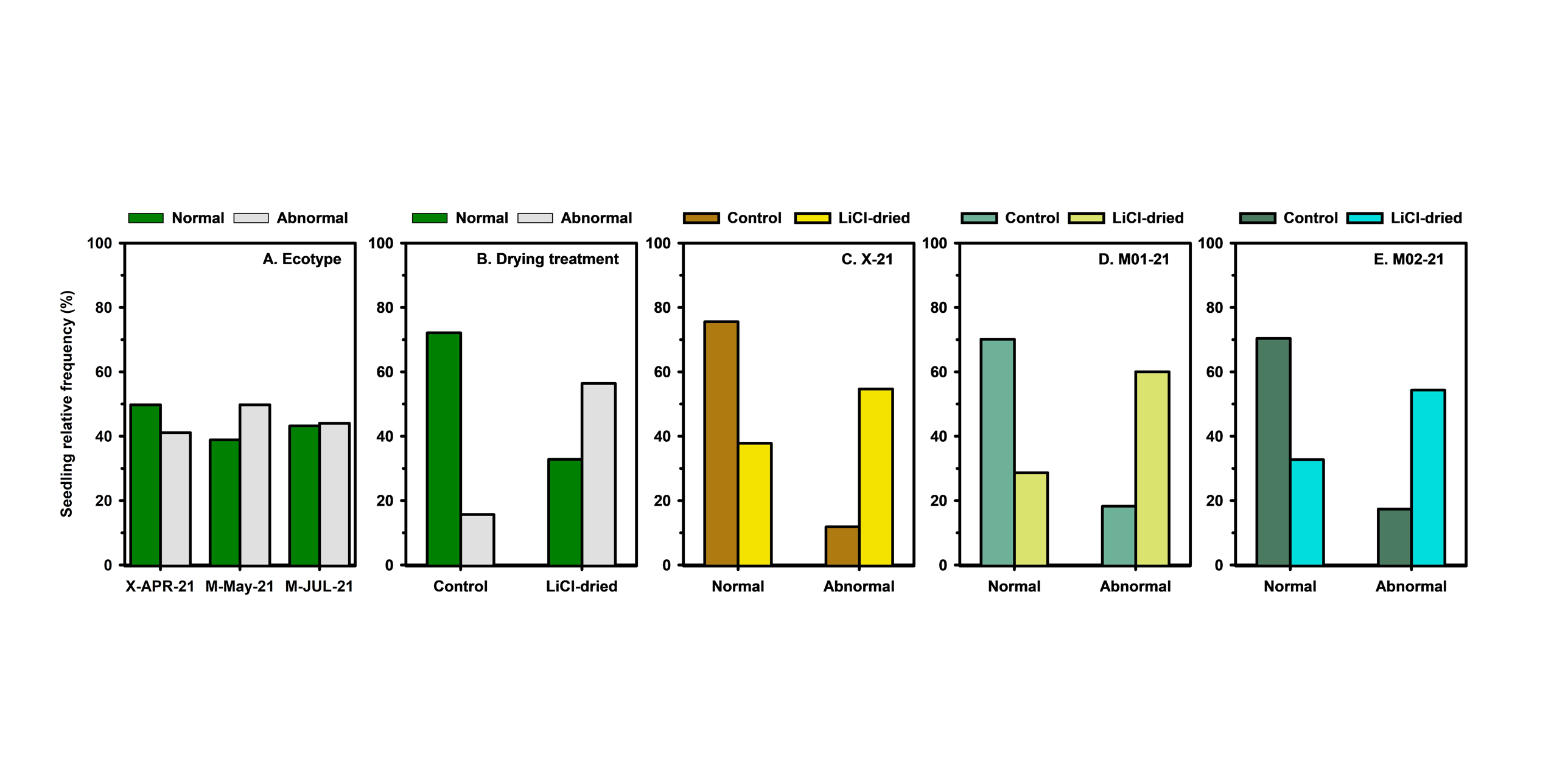

Supplement: S3 Fig — Relative frequency of normal (green bars) and abnormal (gray bars) wiregrass seedlings grouped by (A) ecotype of seed collections (xeric = X-21; mesic = M01-21, M02-21) and (B) drying treatments (control or LiCl-dried) for seeds collected in 2021. Panels C-D show seedling frequencies grouped by drying treatments and seedling status controlling for ecotypes. In (C) brown and yellow bars denote control and LiCL-dried treatments, respectively. Accordingly, in (D) light green bars = control and chartreuse bars = LiCl-dried seeds, and in (E) dark green bars = control and aqua bars = LiCl-dried seeds. Seedling frequencies are reported on a viable seed basis. (JPG) [file pone.0326596.s003.JPG]

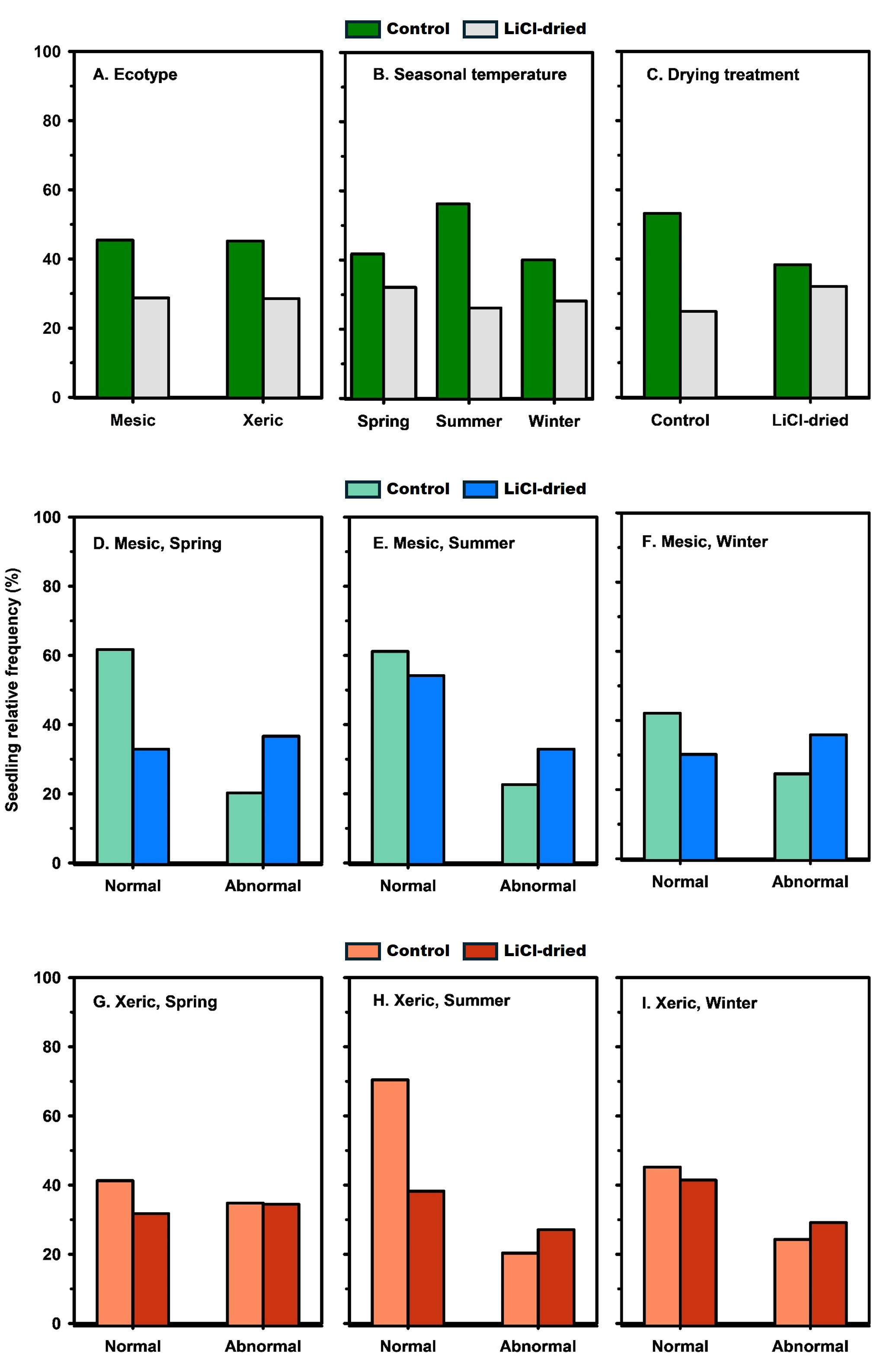

Supplement: S4 Fig — Relative frequency of normal (green bars) and abnormal (gray bars) wiregrass seedlings grouped by (A) ecotype of seed collections (xeric, mesic), (B) simulated seasonal germination temperature (spring = 28/15°C, summer = 35/25°C, winter = 21/8°C), and (C) drying treatments (control or LiCl-dried) for seeds collected in 2022. Panels D-I show frequencies grouped by drying treatments, ecotypes, and simulated seasonal temperatures. In D-F light green and blue bars denote control and LiCL-dried treatments, respectively. In G-I orange and red bars denote control and LiCl-dried treatments, respectively. Seedling frequencies are reported on a viable seed basis. (JPG) [file pone.0326596.s004.JPG]
