# Supplementary material for: Desiccation tolerant yet short-lived seeds: A conundrum for post-harvest handling of a high restoration value bunchgrass?
Source: PLoS One. 2025 Jun 20;20(6):e0326596. doi: 10.1371/journal.pone.0326596 (PMC12180627; doi:10.1371/journal.pone.0326596)
Supplement: S1 Table — Wiregrass seed collection sites, with names used in the manuscript, and type of habitat where plants occurred. Sites M01-21 and M02-21 represent different burn units separated by about 2 km. (PDF) [file pone.0326596.s005.pdf]

Collection Year	Site	Code	Ecotype
2021	Hal Scott Preserve	M01-21	Mesic
	Hal Scott Preserve	M02-21	Mesic
	Econfina Creek	X-21	Xeric
2022	Austin Cary Forest	M-22	Mesic
	Ordway-Swisher Biological Station	X-22	Xeric
