# Supplementary material for: Desiccation tolerant yet short-lived seeds: A conundrum for post-harvest handling of a high restoration value bunchgrass?
Source: PLoS One. 2025 Jun 20;20(6):e0326596. doi: 10.1371/journal.pone.0326596 (PMC12180627; doi:10.1371/journal.pone.0326596)
Supplement: S2 Table — Final Cox regression models for germination of wiregrass seeds collected in 2021 and 2022. The model for germination in 2022 contains the time-dependent covariate Seasonal temperature × Days to account for non-proportionality of the germination response to seasonal temperatures. (PDF) [file pone.0326596.s006.pdf]

Year	Model effects	df	Wald χ^2	*p*
2021	Ecotype	2	9.35	0.0099
	Drying treatment	1	34.89	< 0.0001
	Ecotype × drying treatment	2	6.74	0.0136
2022	Ecotype	1	0.87	0.3509
	Seasonal temperature	2	101.71	< 0.0001
	Drying treatment	1	0.96	0.3278
	Ecotype × Seasonal temperature	2	0.39	0.8247
	Ecotype × Drying treatment	1	0.00	0.9867
	Seasonal temperature × Drying treatment	2	0.54	0.7627
	Ecotype × Seasonal temperature × Drying treatment	2	0.90	0.6386
	Seasonal temperature × Days	1	86.34	< 0.0001
